# Diverse cloud and aerosol impacts on solar photovoltaic potential in southern China and northern India

**DOI:** 10.1038/s41598-022-24208-3

**Published:** 2022-11-16

**Authors:** Jiangyan Yang, Bingqi Yi, Shuai Wang, Yushan Liu, Yuxiao Li

**Affiliations:** School of Atmospheric Sciences and Guangdong Province Key Laboratory for Climate Change and Natural Disaster Studies, Sun Yat-sen University, and Southern Marine Science and Engineering Guangdong Laboratory (Zhuhai), Zhuhai, China

**Keywords:** Atmospheric science, Photovoltaics

## Abstract

Cloud and aerosol are two important modulators that influence the solar radiation reaching the earth’s surface. It is intriguing to find diverse impacts of clouds and aerosols over Southern China (SC) and Northern India (NI) which result in remarkable differences in the plane-of-array irradiance (POAI) that signifies the maximum available solar photovoltaic potential by combining the latest satellite retrieval results and modeling tools. By separating the impacts of cloud and aerosol on the POAI, it is found that clouds are responsible for the most reduction of POAI in the SC, while aerosols and clouds are equally important for the NI region. The frequent occurrences of low and middle level clouds with high optical depth in the SC, as compared with the much lower occurrences of all levels of clouds with lower optical depth in the NI, is regarded as the major reason for the differences in the POAI. The differences in the main compositions of aerosols in the SC (sulfate) and the NI (dust) could be essential to answer the question of why higher aerosol optical depth in the SC whereas leads to weaker reduction in the POAI than that in the NI. The mitigation measures targeting on the controls of different types of aerosols should be considered for different regions.

## Introduction

The research and use of clean and renewable energy have become an important issue especially for the developing countries to meet the need for the rapid population and economic growth^1^. Solar energy is very attractive because it is not only clean but also sustainable. Previous research shows that the total energy received on the Earth in one hour is higher than the total energy consumption in the entire world in a year^2^. Among the several ways to convert and use solar energy, the solar photovoltaic (PV) is a highly efficient and economic option. In the past decades, several developing countries have vigorously promoted the development of solar PV power generation in response to climate change and environmental protection^3^. The global installed solar PV capacity increased from 5.1 to 227.0 GW from 2003 to 2015, and it is expected that the growth rate will continue to increase due to the improvements in the technical and economic factors of PV power generation^4^.

Although solar energy has many advantages, its practical application as a clean energy source is subject to several constraints and key factors modulating the distribution and intensity of the surface solar radiation, such as the daily and seasonal variations of the sun and the meteorological conditions, leading to variations or interruptions in solar PV power generation^5,6^. Solar radiation is attenuated by the atmospheric components, such as the clouds, aerosols, water vapor, carbon dioxide, and ozone, as it is transmitted through the atmosphere. Clouds and aerosols are found to be responsible for the perturbations of the solar radiation reaching the surface which result in the “global brightening and dimming” at different periods of time^7,8^.

As one of the largest modulators of solar radiation at the surface, clouds and their properties can significantly affect surface solar radiation^9,10^. Hatzianastassiou et al.^11^ found that the decrease in the low cloud amount can explain up to 70% of the increasing trend in the global surface solar radiation from 1990 to 2000. From ground-based observations, Xia^12^ found that the interannual variation of sunshine hour in China shows a strong negative correlation with the interannual variation of low cloud amount. Dumka et al.^13^ studied the impacts of aerosol and cloud on the solar energy potential over the central Gangetic Himalayan region and emphasized the much larger impacts of cloud than aerosol.

Aerosols also play an important role in attenuating solar radiation directly by scattering and absorbing processes, and by acting as cloud condensation nuclei to influence radiation indirectly^14–16^. Qian et al.^17^ found that air pollution is the cause of reduction in the surface solar radiation from 1954 to 2001 although the number of clear-sky days in China increases. It is also found that the anthropogenic aerosol emissions contribute to the reduced surface solar radiation from 1971 to 2002 in the European region in addition to the impacts of clouds^18^. Fei et al.^19^ found that despite the increases in the total cloud cover, low cloud cover, and water vapor content, the surface solar radiation over the Taklamakan desert still increases due to the decrease of dust aerosols from 1980 to 2009. Li et al.^20^ presented a detailed study emphasizing the reduction of solar photovoltaic resources by air pollution in China. Li et al.^21^ pointed out that the changes in aerosol properties lead to the increase of surface solar irradiance in east China in the past decade. The study by Sweerts et al.^22^ suggested strict air pollution control and reduced fossil fuel consumptions could contribute to the increase in the surface radiation. Shi et al.^23^ attributed the reasons for the surface brightening in eastern and central China to the reduction of air pollution since 2013. Wang et al.^24^ revealed that the underestimation of anthropogenic aerosol emissions could be the reason for the low bias of surface solar radiation in the Coupled Model Intercomparison Project Phase 6 results.

**Figure 1:**
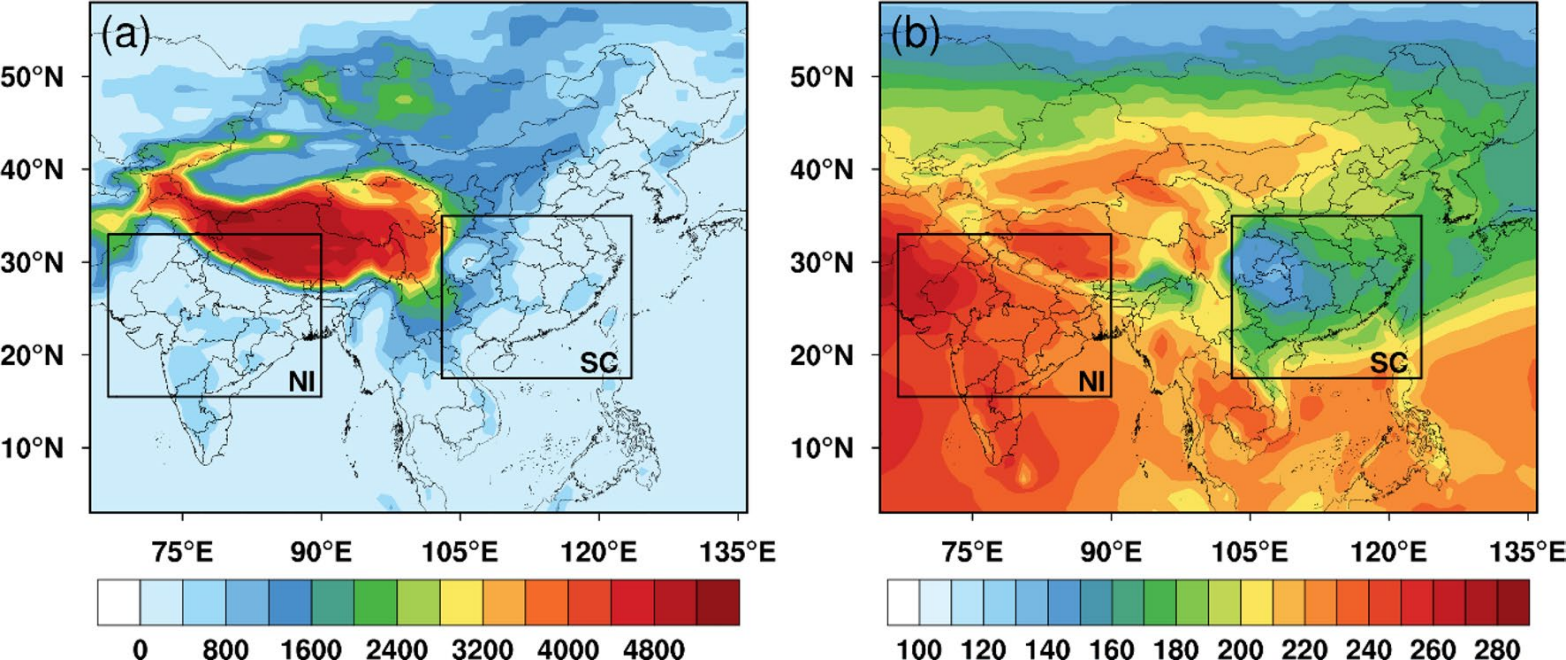
The (**a**) surface altitude (Unit: m) and (**b**) annual averaged downward shortwave irradiance at the surface (Unit: W/m^2^) over southern China and northern India from 2003 to 2019 based on the CERES SYN1deg data product. The map was generated by ESRI ArcGIS 10.5 software available at the ESRI website (https://www.esri.com/en-us/arcgis/products/arcgis-platform/overview). The administrative boundary shapefile for southern China is available at the RESDC website (https://www.resdc.cn/) and the administrative boundary shapefile for northern India is available at the GADM website (https://gadm.org/download_country.html).

From the above analysis, it is known that clouds and aerosols both exert remarkable impacts on the surface solar irradiance. However, the studies that quantify the influences of clouds and aerosols simultaneously are lacking. Furthermore, the relative importance of clouds and aerosols on the specific applications of solar energy, such as the solar PV, is still not clear. We notice an interesting phenomenon that the solar irradiances at the surface in southern China and northern India are very different although the two regions locate at the similar latitude ranges (Fig. 1), based on the CERES (Clouds and the Earth’s Radiant Energy System) SYN1deg data product. This study aims at finding the reasons for the different reductions of incoming surface solar radiation, and quantifying the different impacts of aerosols and clouds on the solar PV potential in the two regions.

## Data and Methods

### Data

The Clouds and the Earth’s Radiant Energy System (CERES) is part of the NASA Earth Observing System and provides the radiative fluxes at the top and bottom of the atmosphere under different conditions for studies about the Earth’s radiation budget^25^. In this study, the CERES SYN1deg (Synoptic) Edition 4A products which include the surface shortwave radiant fluxes at the spatial resolution of 1° × 1° and the temporal resolution of 3-hourly are used^26,27^. Specifically, the direct and diffused components of the surface shortwave radiant fluxes under four conditions: (1) all-sky: including aerosols and clouds; (2) clear-sky: including aerosols without clouds; (3) all-sky-no-aerosol: including clouds without aerosols; and (4) pristine: no aerosols and no clouds, are derived. Additionally, the CERES-MODIS cloud area fraction (CF), cloud optical depth (COD), as well as the aerosol optical depth (AOD) from 2003 to 2019 accompanying with the CERES SYN1deg dataset are collected and used.

The Modern-Era Retrospective analysis for Research and Applications Version 2 (MERRA-2) is a global reanalysis data set that featuring the assimilation of multi-source observations of aerosols^28^. The MERRA-2 data set also provides surface solar shortwave radiant fluxes under four different conditions (i.e., all-sky, clear-sky, all-sky-no-aerosol, and pristine). The different types of aerosols in MERRA-2 including dust, sulfate, black carbon, organic carbon, and sea salt, are simulated by the GOCART (Goddard Chemistry, Aerosol, Radiation and Transport) model which considers multiple processes that influence aerosol emission and distribution^29–31^. A comparison of the aerosol optical depth from MERRA-2 and the AERONET (Aerosol Robotic Network) for the period of 1980–2016 shows that the two data sets are in good spatial agreement on a global scale^32^. In this study, the monthly averaged radiative flux at the surface and the AOD of five types of aerosol (i.e., dust, black carbon, organic carbon, sea salt, and sulfate) at the spatial resolution of 0.5° × 0.625° are derived from the MERRA-2 reanalysis data set. We also carry out some comparisons between the MERRA-2 and CERES SYN1deg data sets before they were used to interpret the different PV potentials over the NI and SC regions (see the Supplementary material).

### Model description

The PVLIB-Python (version 0.7.2) is an open-source software developed by Sandia National Laboratory, which provides a complete set of advanced modeling tools for evaluating the solar PV potential and for modeling the performances of various PV systems with different degrees of complexities. The model takes the observed or simulated direct and diffused components of the surface solar irradiances and the atmospheric conditions as inputs, and outputs the maximum potential irradiance that could be transformed into electric power, as well as the total electric power estimated with various system settings^3,10,33,34^.

This study uses the above-mentioned surface radiant fluxes from the CERES SYN1deg data set as the input variables to the PVLIB model and outputs the calculated plane-of-array-irradiance (POAI) which represents the maximum theoretical potential of PV power generation. The POAI is the solar irradiance incident on the plane of PV panel, which is measured in the unit of kWh/m^2^, and it is closely related to the position of the sun, the orientation of the PV panel array, as well as the other factors that influence the direct and diffused components of incident irradiance, such as the surface reflectance, shading, and aerosols and clouds. Note the POAI in this study refers to the total POAI accumulated during the daytime of the day, and thus is measured in the unit of kWh/m^2^/d. The modeling of POAI by the PVLIB-python model generally involves the conversion of the direct and diffused solar irradiances into the direct normal irradiance (DNI) and diffuse horizontal irradiance (DHI), and further calculates POAI from the DNI and DHI^35^. In this study, we only consider the panel of PV system with fixed tilting angle, where the optimal tilting angle ($\it \uptheta$) is selected following the study by Landau^36^ as follow:

1 $$\theta = \left\{ {\begin{array}{*{20}l} {\varphi \times 0.87, } \hfill & {\varphi < 25^{^\circ } } \hfill \\ {\varphi \times 0.76 + 3.1,} \hfill & {25^{^\circ } \le \varphi \le 50^{^\circ } } \hfill \\ {\varphi ,} \hfill & {\varphi > 50^{^\circ } } \hfill \\ \end{array} } \right.,$$

where $\varphi$ denotes the latitude of the PV system in degrees.

### Methodology and experiment design

As illustrated above, the CERES SYN1deg data set already provides the surface shortwave fluxes under all-sky (AS, with clouds and aerosols), clear-sky (CS, with aerosols but without clouds), all-sky-no-aerosol (NA, with clouds but without aerosols), and pristine (PS, without clouds and aerosols) conditions. It is straightforward to calculate the corresponding POAI under the different conditions with the PVLIB-python package. As a result, the reduction in POAI due to the impacts of clouds can be defined and calculated as the cloud effect (CE) as follows:

2 $${\text{CE}} = {\text{POAI}}_{{{\text{CS}}}} - {\text{POAI}}_{{{\text{AS}}}} ,$$

where POAI_CS_ and POAI_AS_ denote the POAI under the clear-sky and all-sky conditions, respectively. Similarly, the impacts of aerosols on POAI reduction can be defined and calculated as the aerosol effect (AE):

3 $${\text{AE}} = {\text{POAI}}_{{{\text{NA}}}} - {\text{POAI}}_{{{\text{AS}}}} ,$$

where POAI_NA_ denotes the POAI under the all-sky-no-aerosol condition.

Meanwhile, it is also helpful to calculate the percentages of reductions in the all-sky POAI contributed by clouds (PCE) and aerosols (PAE) which could clearly show the relative importance of the two factors. The PCE and PAE are calculated as dividing the CE and AE by the POAI under all-sky condition respectively as follows:

4 $${\text{PCE}} = \frac{{\text{CE }}}{{{\text{POAI}}_{{{\text{AS}}}} }}*100\% ,$$

5 $${\text{PAE}} = \frac{\text{AE}}{{{\text{POAI}}_{{{\text{AS}}}} }}*100\% .$$

Specifically, we define the aerosol effect efficiency (AEE) in the way similar as the aerosol radiative forcing efficiency^37^ to illustrate the capacities of different types of aerosol in the reduction of solar PV potential (which is the POAI) per unit AOD:

6 $${\text{AEE}} = \frac{{{\text{AE}}}}{{{\text{AOD}}_{550} }},$$

where AOD_550_ is the aerosol optical depth at the wavelength of 550 nm, and the unit of AEE is kWh/m^2^/d/τ.

### The areas of interests

This study focuses on two regions over southern China and northern India. Previous studies show that China and India are both rich in solar energy resources. For example, up to 67% of the total area in China has abundant solar energy with more than 2200 sunlight hours and more than 5000 MJ/m^2^ of the annual irradiance amount^38^. India generally has 250–300 sunny days in a year and the average solar radiation intensity is 200 MW/km^2^^39^. It is known that the geographical location and altitude are critical factors to determine the solar energy resources. But it is striking to find that the southern China (17° N–35° N, 103° E–124° E) and the northern India (15° N–33° N, 67° E–90° E) within the similar latitude and altitude ranges (Fig. 1a) exhibit large differences in the ground surface shortwave irradiances (Fig. 1b), given the facts that both areas are similar in size and in the population density^40^. In addition, both areas suffer from severe haze and air pollution problems^41,42^.

## Results

### Spatial and temporal distributions of POAI over the SC and NI

**Figure 2:**
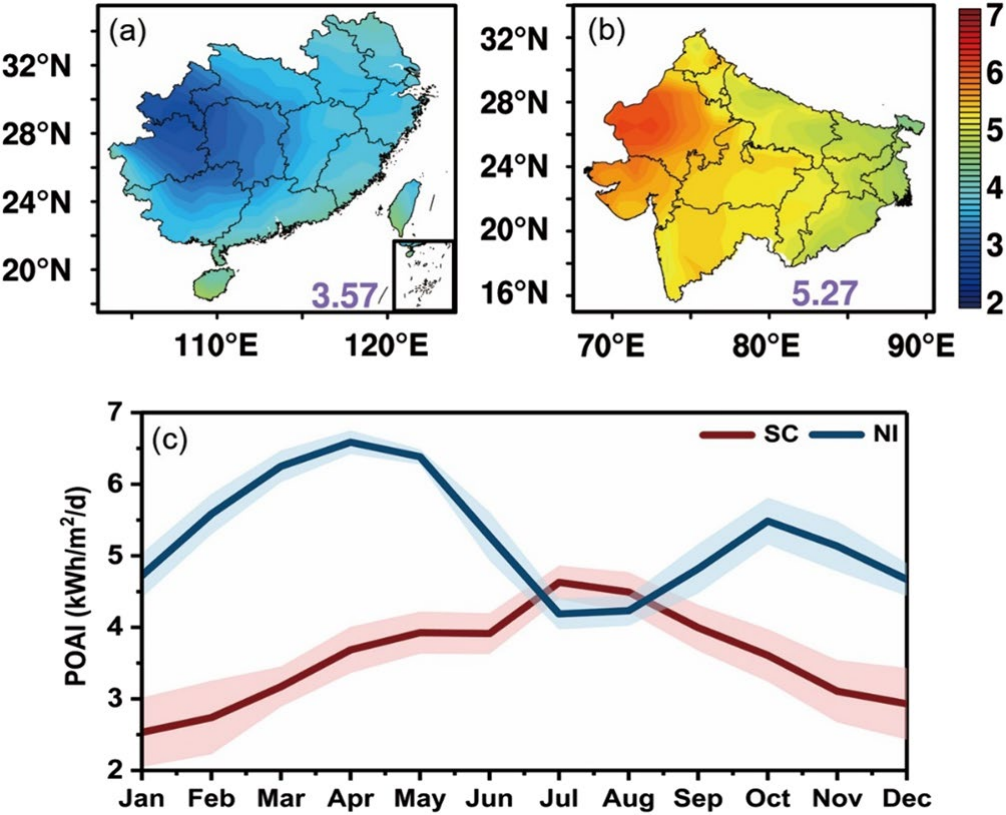
The annual averaged POAI over (**a**) the SC and (**b**) the NI under the all-sky condition from 2003 to 2019 (unit: kWh/m^2^/d). The numbers represent the values of regional averaged POAI; (**c**) the monthly averaged POAI over the SC and NI. The color shadings denote the standard deviations of POAI. The map was generated by ESRI ArcGIS 10.5 software available at the ESRI website (https://www.esri.com/en-us/arcgis/products/arcgis-platform/overview). The administrative boundary shapefile for southern China is available at the RESDC website (https://www.resdc.cn/) and the administrative boundary shapefile for northern India is available at the GADM website (https://gadm.org/download_country.html).

Figure 2a,b show the annual averaged solar PV potential over the SC and NI regions in terms of POAI under all-sky condition. It is apparent that the two regions show large differences in the solar PV potential (3.57 kWh/m^2^/d in the SC vs. 5.27 kWh/m^2^/d in the NI). The spatial distribution of POAI in the two regions are also different. In the SC region, the inland area has much lower POAI than the coastal area. A low POAI center (lower than 4 kWh/m^2^/d) locates at the southeastern edge of the Sichuan basin in China and covers Guizhou, Chongqing, and Hunan. The NI region is characterized with more abundant solar PV resources in the west than the east. The Rajasthan state (23° N–30° N, 39° E–78° E) possesses the highest PV potential (~ 6.0 kWh/m^2^/d) in the NI and it is also recognized as the area with the highest annual radiation output over the globe^43^. Figure 2c shows the variations of monthly averaged all-sky POAI over the SC and the NI regions. It is found that the solar PV potential over the NI is almost always higher than that of the SC except in July and August. In the SC region, the POAI reaches the peak value in July and the lowest value in January. On the contrary, the POAI in the NI region shows two peaks in April and October, and is lowest in January and July. Thus, the difference in the POAI between the two regions is the largest in spring (March) when the POAI in the NI is twice as large as that in the SC, but the lowest in summer (August) which is only about 0.25 kWh/m^2^/d. Several factors including clouds, aerosols, water vapor, and ozone are considered as the most important modulators of solar resources. To better explain this phenomenon, we try to explore the driving factors of the surface solar PV potential in the next sections.

### The impacts of clouds and aerosols on POAI over the SC and NI

Li et al.^20^ pointed out the important role of aerosols on the reduction of POAI which is comparable to the contribution of clouds in China. In this section, we attempt to separate the impacts of clouds and aerosols on POAI following Li et al. (2017) in the SC and the NI regions for better understanding of the spatial and temporal distributions of POAI. The reduction in POAI due to the impacts of clouds and aerosols are defined as the cloud effect (CE) and aerosol effect (AE) in the unit of kWh/m^2^/d, respectively. The percentages of reductions in the all-sky POAI contributed by clouds (PCE) and aerosols (PAE) are also calculated to show the relative importance of the two factors.

**Figure 3:**
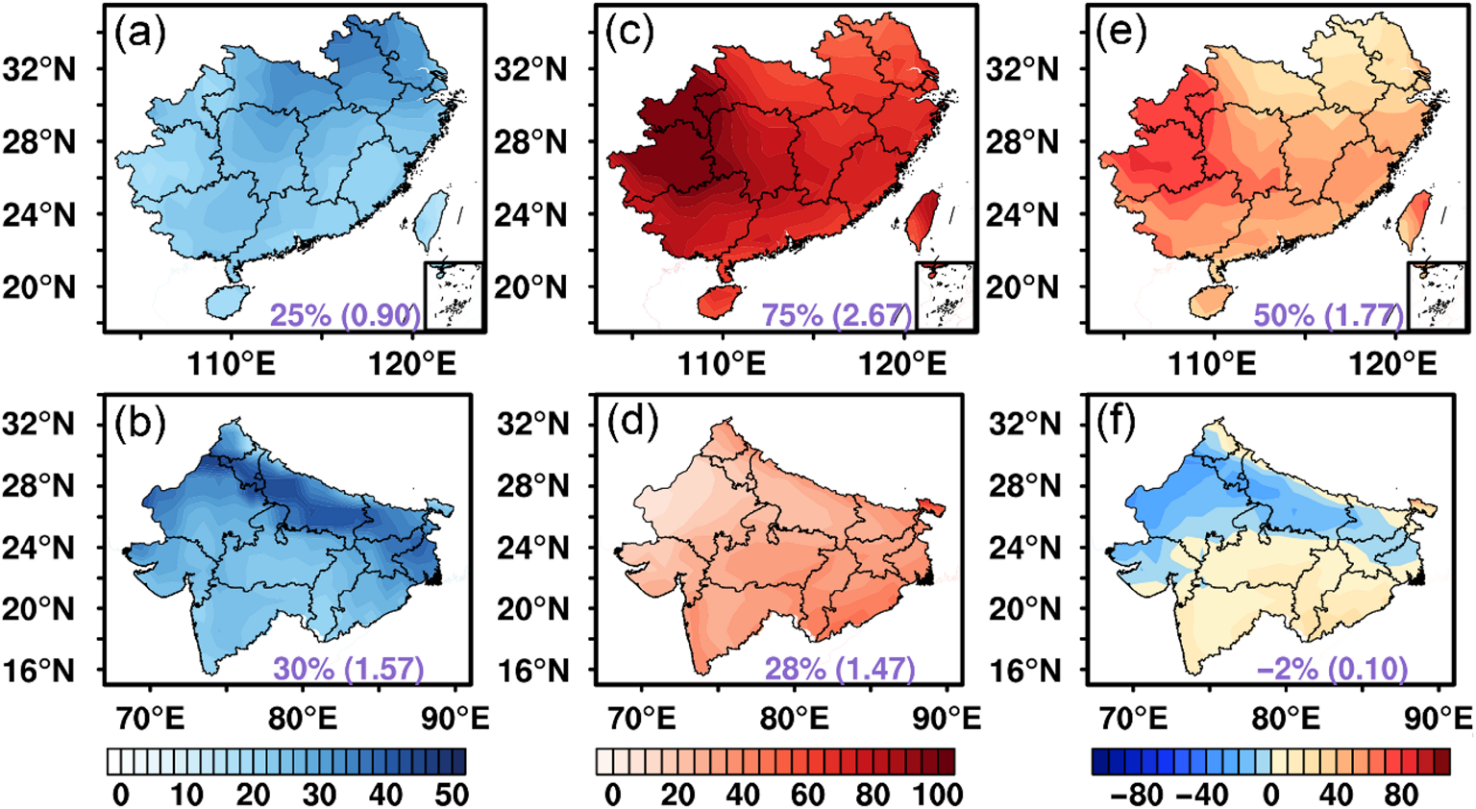
The effects of aerosols and clouds on POAI over the SC (top panels) and the NI (bottom panels): (**a**,**b**) PAE; (**c**,**d**) PCE; (**e**,**f**) PCE minus PAE (Unit: %). The numbers outside the parentheses within each panel denote the regional averaged PAE and PCE, while the numbers inside the parentheses represent the regional averaged AE and CE (Unit: kWh/m^2^/d). The map was generated by ESRI ArcGIS 10.5 software available at the ESRI website (https://www.esri.com/en-us/arcgis/products/arcgis-platform/overview). The administrative boundary shapefile for southern China is available at the RESDC website (https://www.resdc.cn/) and the administrative boundary shapefile for northern India is available at the GADM website (https://gadm.org/download_country.html).

Figure 3 shows the impacts of clouds and aerosols on the POAI in the SC and NI regions in terms of the PAE and PCE, as well as the result of PCE minus PAE which represents the relative importance of clouds to aerosols. In the SC region, the POAI is reduced by 0.9 kWh/m^2^/d due to aerosols, which amounts to 25% of the all-sky POAI. The northern part of the SC is more affected by aerosols than the southern part. For the NI region, the POAI reduction due to aerosols increases to 1.57 kWh/m^2^/d or about 30% of the local all-sky POAI. Similar to the SC, stronger PAE is found in the northern part of the NI than that in the southern part, except that the north–south contrast is stronger in the NI. This is likely due to the dense population and severe air pollution at the Indo-Gangetic Plain (IGP, 24° N–31° N, 74° E–90° E).

The impact of clouds on the POAI in the SC is up to 2.67 kWh/m^2^/d which constitutes 75% of the POAI under all-sky condition. The strongest cloud effect lies to the western part of the SC in Guizhou and Chongqing. In contrast, the magnitude of POAI reduction by clouds is about equal to that of aerosols over the NI region. The overall distribution of PCE in the NI features slightly higher PCE in the southern NI than the northern NI. The Rajasthan state locating at the northwestern part of India (the Thar desert) is the location where the lowest PCE is found.

By subtracting the PCE by the PAE, it is straightforward to figure out that the dominant driving factors on the POAI in the two regions are different (Fig. 3e,f). In the SC, the PCE is predominantly higher than the PAE. In addition, the cloud effect is growingly important from north to south and from east to west over the SC region. On the other hand, the NI region experiences relatively equal reductions of the POAI by clouds and aerosols. It is also noted that the aerosol effect plays the overarching role in the northern NI region.

**Figure 4:**
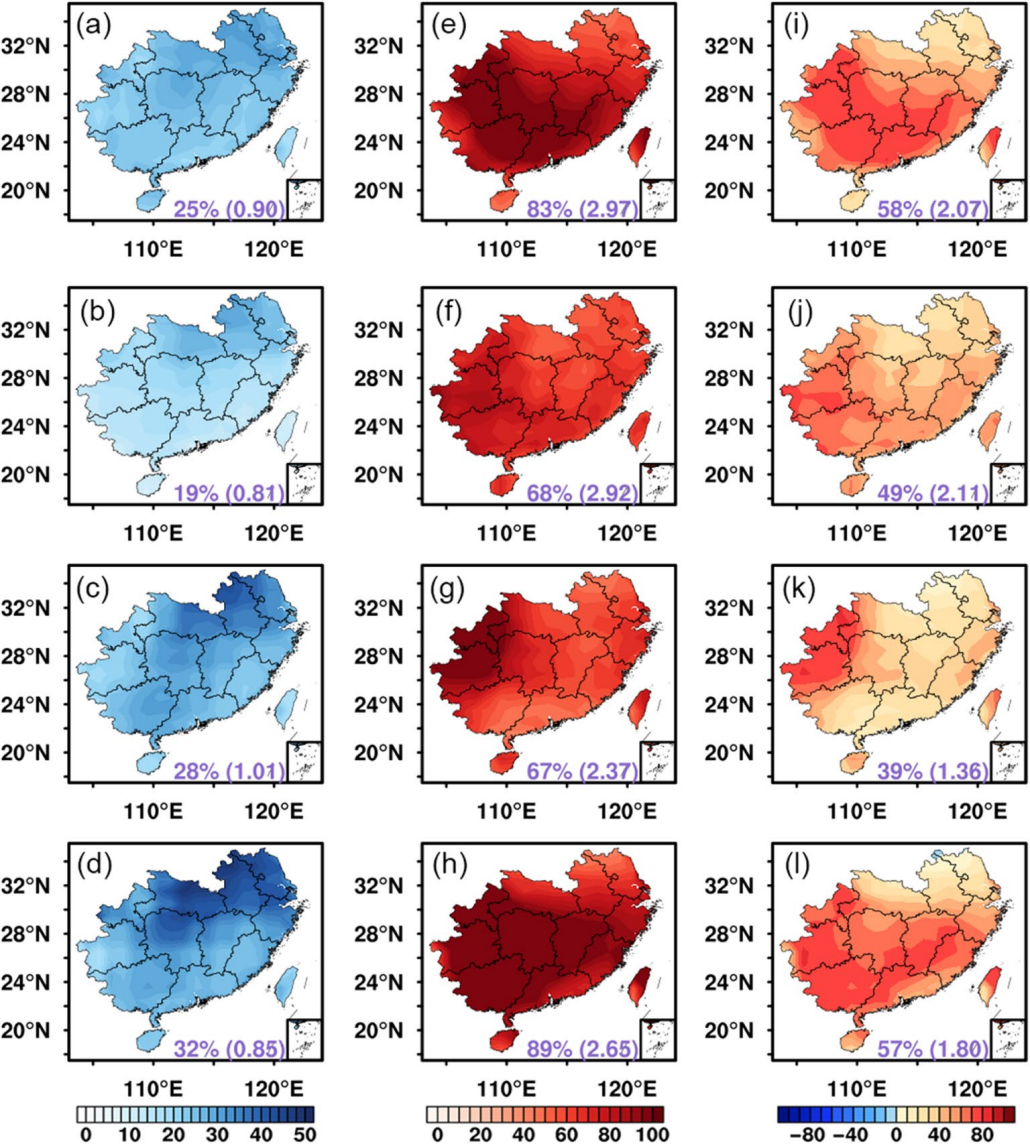
Seasonal averaged impacts of aerosols and clouds on POAI over the SC from 2003 to 2019: (**a**–**d**) PAE; (**e**–**h**) PCE; (**i**–**l**) PCE minus PAE (Unit: %). (**a**,**e**,**i**) spring (March–April–May); (**b**,**f**,**j**) summer (June–July–August); (**c**,**g**,**k**) autumn (September–October–November); (**d**,**h**,**l**) winter (December–January–February). The numbers outside the parentheses represent the regional averaged PAE or PCE, while the numbers inside the parentheses represent the regional averages of AE or CE in the unit of kWh/m^2^/d. The map was generated by ESRI ArcGIS 10.5 software available at the ESRI website (https://www.esri.com/en-us/arcgis/products/arcgis-platform/overview). The administrative boundary shapefile for southern China is available at the RESDC website (https://www.resdc.cn/).

**Figure 5:**
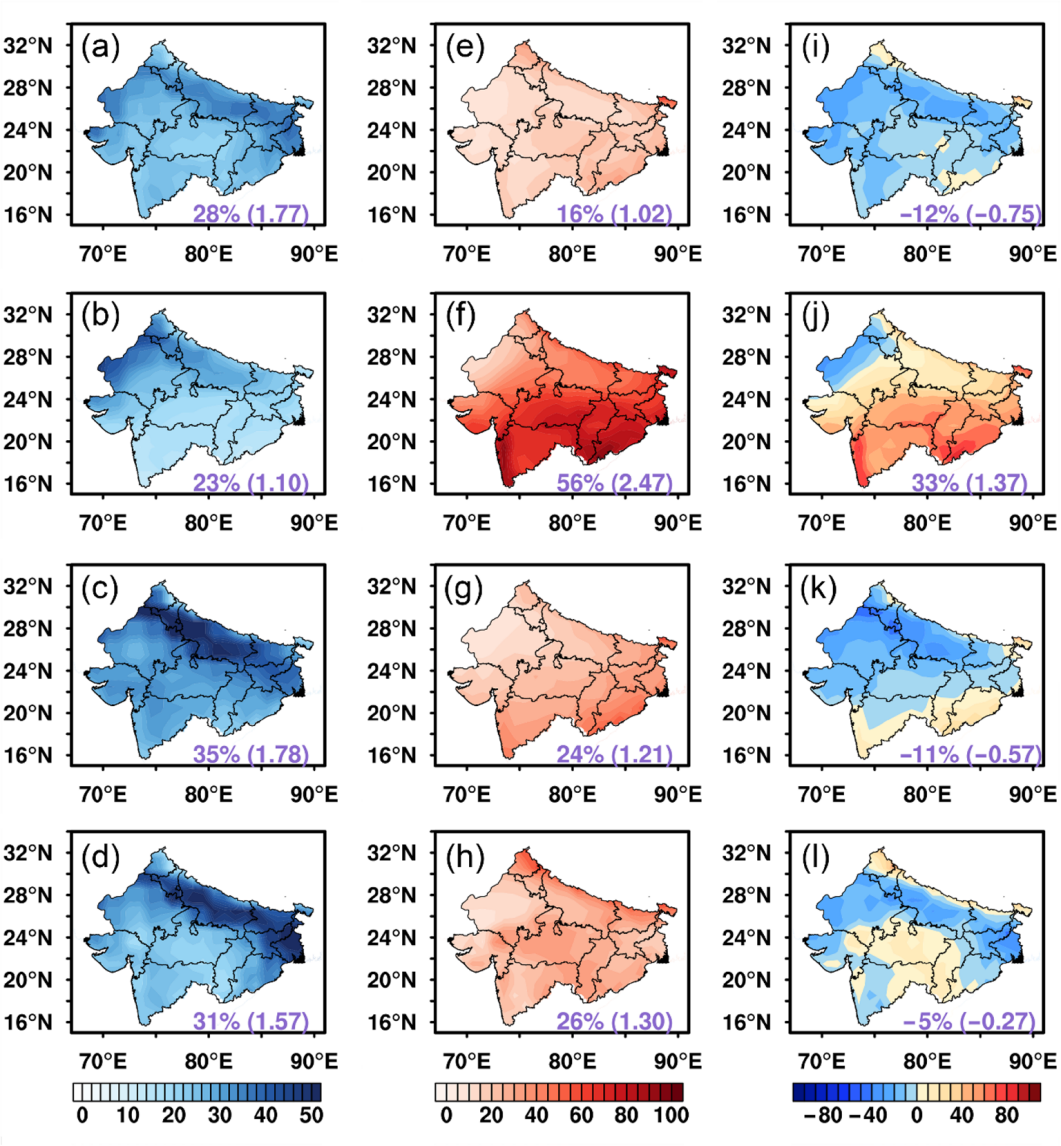
The same as Fig. 4, but for the NI. The map was generated by ESRI ArcGIS 10.5 software available at the ESRI website (https://www.esri.com/en-us/arcgis/products/arcgis-platform/overview). The administrative boundary shapefile for northern India is available at the GADM website (https://gadm.org/download_country.html).

The seasonal variations of the impacts of clouds and aerosols over the SC and NI regions are provided in Figs. 4 and 5, respectively. Note for the ease of comparison between the SC and NI regions, the four seasons are divided as spring (March–April–May), summer (June–July–August), autumn (September–October–November), and winter (December–January–February) in this study, although it is more typical to divide three seasons (pre-monsoon, monsoon, and winter) in the Indian region. It is evident to find that the cloud and aerosol impacts on the POAI vary significantly with seasons and locations. In the SC region, the PAE is found to be the largest in winter and the lowest in summer. The PAE is always higher in the northern SC than the southern SC, while the north–south contrast is the lowest in summer and the largest in winter. Note in terms of AE, the regional averaged AE is highest in autumn (1.01 kWh/m^2^/d) instead of winter, because the PAE only exhibits the relative contribution of aerosols which is also modulated by the values of the all-sky POAI. As a result, higher (lower) PAE does not necessarily mean higher (lower) AE. For the impacts of clouds, the PCE is the highest in winter, follows by spring and summer, and is the lowest in autumn, while the largest CE is found in spring and summer. Geographically, the northeastern part of the SC region is less affected by clouds, while the southern and especially the southwestern SC are the most-affected areas by clouds with PCE larger than 80%. The results clearly show that the CE is almost three times larger than the AE in each season except for autumn. The monthly variations of AE and CE (Fig. S1) confirm that cloud is the major factor affecting the solar PV potential in the SC at all seasons.

In the NI region, the PAE is larger than 30% in autumn and winter, although the AE is the largest in spring and autumn. Similar as the SC region, the northeast part of the NI always experiences larger aerosol impacts than the southern NI. But it is found that the regional averaged AE in the NI almost doubles the value of the SC region except in summer, although the PAEs in both regions are similar. The PCE in the NI varies dramatically from the lowest value in spring (16%) to the highest in summer (56%), and remains at about 24–26% in autumn and winter. It is also generally found that the PCE is relatively smaller in the northern NI than in the central and southern NI, and the northwestern NI is the area the least affected by clouds. The CE values in the NI region, however, are typically about half of the CE values in the SC region except in summer. As a result, Fig. 5i–l show that the aerosol effect surpasses the cloud effect and play the dominant role in modulating the POAI in the NI except for the southern NI especially in summer. In the northwestern NI (the Rajasthan state), the aerosol effect is strong in all seasons probably due to the persistent impacts of dust aerosols transported from the Thar desert. We further investigate the reasons for the different degrees of cloud effect in the two regions in the next section. It is also interesting to note that the variations of AE and CE are opposite in phase especially for the NI region (Fig. S1). This is indicative of the possible nonlinear aerosol-cloud interactions that are hardly identifiable in this study because the aerosol effect could be somehow masked at the presence of clouds.

### The differences in cloud properties between the SC and NI

In this section, we explore the reasons why the cloud effects in the SC and NI regions are so different at various seasons. As the radiative effect of clouds is closely related to their microphysical and optical properties, the cloud fraction (CF) and cloud optical depth (COD) are shown to provide more insight into this problem.

Figure S2 shows the seasonal averaged cloud area fraction over the SC and NI regions. It is interesting to find that the SC region typically has cloud fraction of up to 0.64–0.77 which is remarkably higher than that over the NI region (0.32–0.37) except for summer (0.77). The western part of the SC almost always has cloud fractions of more than 0.8 all year round. Comparatively, the NI region only has cloud area fraction exceeding 0.8 over the east and southeast part in summer. It is also found that the total COD in the SC region is 2–4 times larger than that over the NI region except for summer, and the areas with high COD and high CF correspond well with each other (Fig. S3). Figure S4 further shows the monthly variations of CF and COD for the low (surface–700 hPa), middle (700–300 hPa), and high (300–50 hPa) clouds as well as the total clouds in the SC and NI regions. Over the SC, the middle cloud (which could be mostly mixed-phase clouds) generally has the largest CF and COD which remain at high values during the whole year. It is found that the variations of CF and COD with time for the low and high cloud are mostly opposite in phase (Fig. S4). The decrease in the COD of middle and low clouds could be the main reason for the high peak of POAI in the summer time in the SC, although the total CF is still large at the same time. Comparatively, the CF of high and middle clouds in the NI starts to increase from May and reach the peak in July, with the COD showing corresponding increase in the same period (the monsoon season). As a result, the increased total CF and COD could be the main reason for the low POAI in summer in the NI. It is certainly worthwhile to further investigate the reasons for the different cloud properties in the two regions in the future studies.

**Figure 6:**
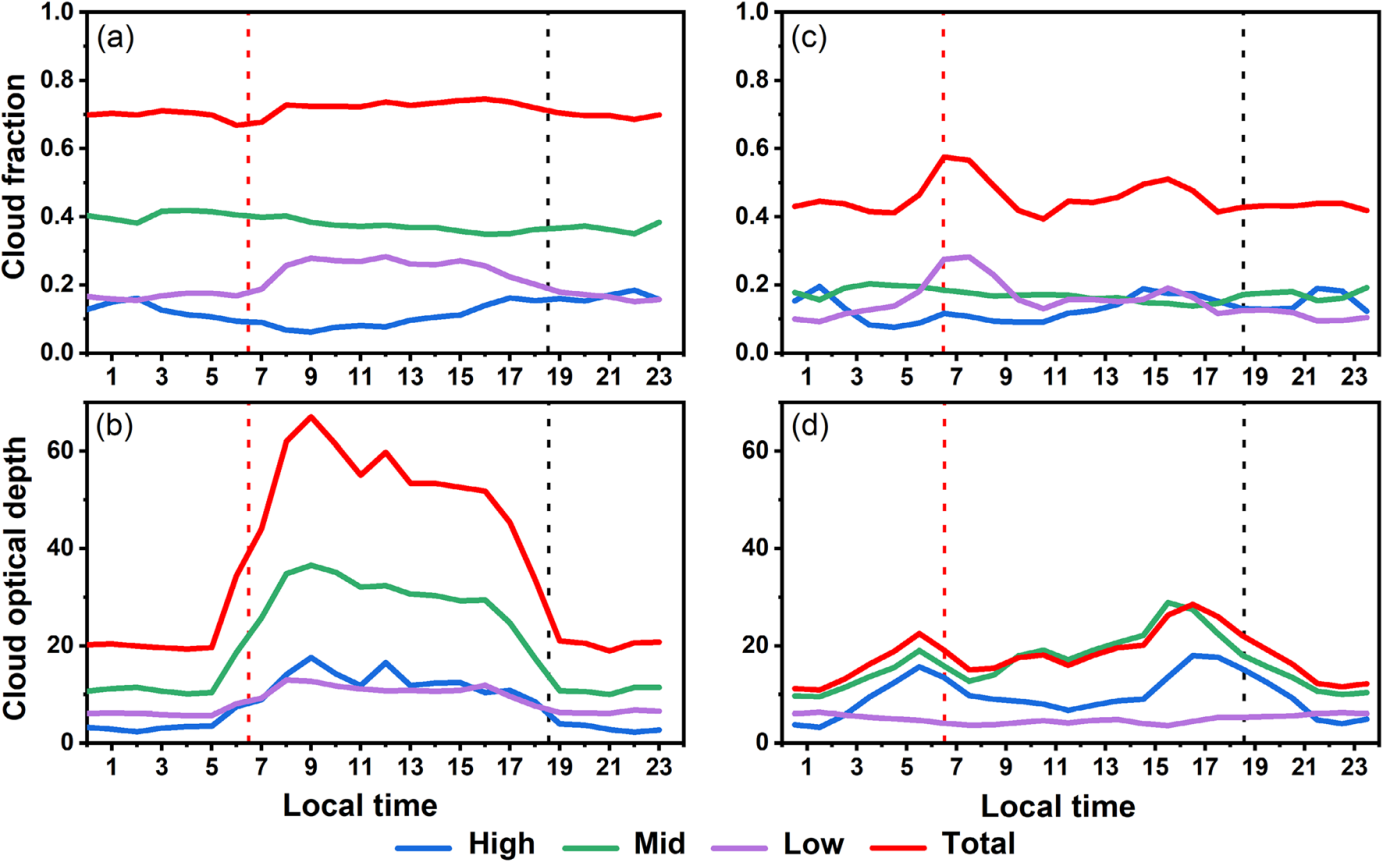
Diurnal variations of (**a**,**c**) cloud fraction and (**b**,**d**) cloud optical depth from 2003 to 2019 over the SC (left column) and NI (right column). Note the cloud optical depth has been weighted by the cloud fraction. The local time for the SC is Beijing time (UTC + 8:00), and the local time for the NI is India standard time (UTC + 5:30). The red and black dotted lines show the averaged sunrise time and the sunset time for the NI and SC regions, respectively.

An interesting point is that the different diurnal variations of CF and COD could be another factor on the different POAI distributions over the SC and NI (Fig. 6). The variations of cloud properties during the daytime are especially important for the solar PV applications. It is evident that although the high cloud CF is low at sunrise, the middle cloud CF remains high and the low cloud CF increases by 0.1 which results in a relatively constant total CF of ~ 0.7 in the SC. The COD of all layers of clouds dramatically increases at sunrise and slightly decreases at sunset. Conversely, although the total CF reaches the maximum (~ 0.6) at sunrise, it fluctuates and decreases to ~ 0.4 at noon and sunset in the NI. The COD of middle and high clouds gradually increases during the day but remains much lower than that over the SC.

### The differences in aerosol properties over the SC and NI

**Figure 7:**
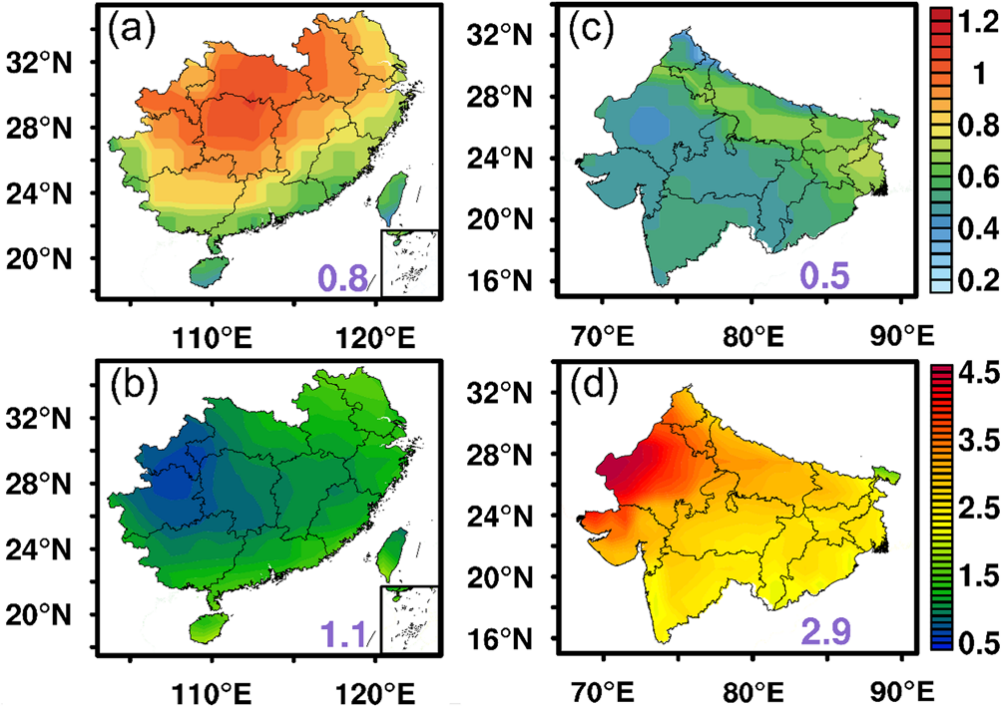
Annual averaged (**a**,**c**) AOD and (**b**,**d**) aerosol effect efficiency (Unit: kWh/m^2^/d/τ) from 2003 to 2019 over the SC and NI. The map was generated by ESRI ArcGIS 10.5 software available at the ESRI website (https://www.esri.com/en-us/arcgis/products/arcgis-platform/overview). The administrative boundary shapefile for southern China is available at the RESDC website (https://www.resdc.cn/) and the administrative boundary shapefile for northern India is available at the GADM website (https://gadm.org/download_country.html).

The dependence of the POAI on the aerosol properties over the SC and NI is investigated in this section. Figure 7a shows that the AOD is the largest in the northern and central SC which ranges from 0.6 to 0.9. For the NI region, the highest AOD is found at the northern part along the southern edges of the Himalayas mountain ranges, with the AOD larger than 0.7 locating over the IGP area (Fig. 7c). It is found that the regional averaged AOD in the SC and NI are comparable, with the AOD in the SC apparently larger than that in the NI. However, the AE (PAE) shows an opposite pattern in that the aerosol impacts on POAI is even larger in the NI than that in the SC (Figs. 4 and 5). This indicates that the aerosols in the two regions could have different capabilities in reducing the surface solar PV potential. The aerosol effect efficiency (AEE) for the two regions are shown in Fig. 7b,d. The results confirm our speculation that the aerosols over the NI are much more efficient than the counterparts over the SC in the extinction of solar radiation. The averaged AEE in the NI is about 2.9 kWh/m^2^/d/τ, as compared with that in the SC at about 1.1 kWh/m^2^/d/τ. We further find that the AEE is highly variable in the NI, where the Rajasthan state and its nearby regions show much higher AEE of up to 4.5 kWh/m^2^/d/τ. The different aerosol types from different sources could be the reason for the different aerosol effect efficiencies.

The aerosol properties and radiative fluxes at the surface within the MERRA-2 data set are used to further illustrate the different impacts of various types of aerosols. Before we use the MERRA-2 products for this purpose, some comparisons of the surface downward shortwave radiative fluxes under all-sky, all-sky-no-aerosol, clear-sky, and pristine conditions are carried out against the CERES SYN1deg products over the two regions (Fig. S5). Figure S5 shows that the surface net downward radiative fluxes under the pristine and clear-sky conditions in both datasets agree well, except that the MERRA-2 fluxes are systematically and slightly larger. The surface shortwave aerosol radiative forcing (Fig. S6), which is calculated as the net downward shortwave radiative flux under the all-sky condition minus that under the all-sky-no-aerosol condition at the surface, again shows reasonable agreement between the two data sets, except that the aerosol radiative forcing of the MERRA-2 product is underestimated. From Fig. S7, the scatter plots show weaker aerosol radiative forcing in the MERRA-2 data set for both regions. It is found that the cloud radiative forcing, however, are not properly accounted for (much weaker) over the NI region in the MERRA-2 product. Previous studies also show that the aerosol properties in the MERRA-2 product are reasonable as compared with the ground-based and satellite-borne aerosol retrievals^44–48^.

**Figure 8:**
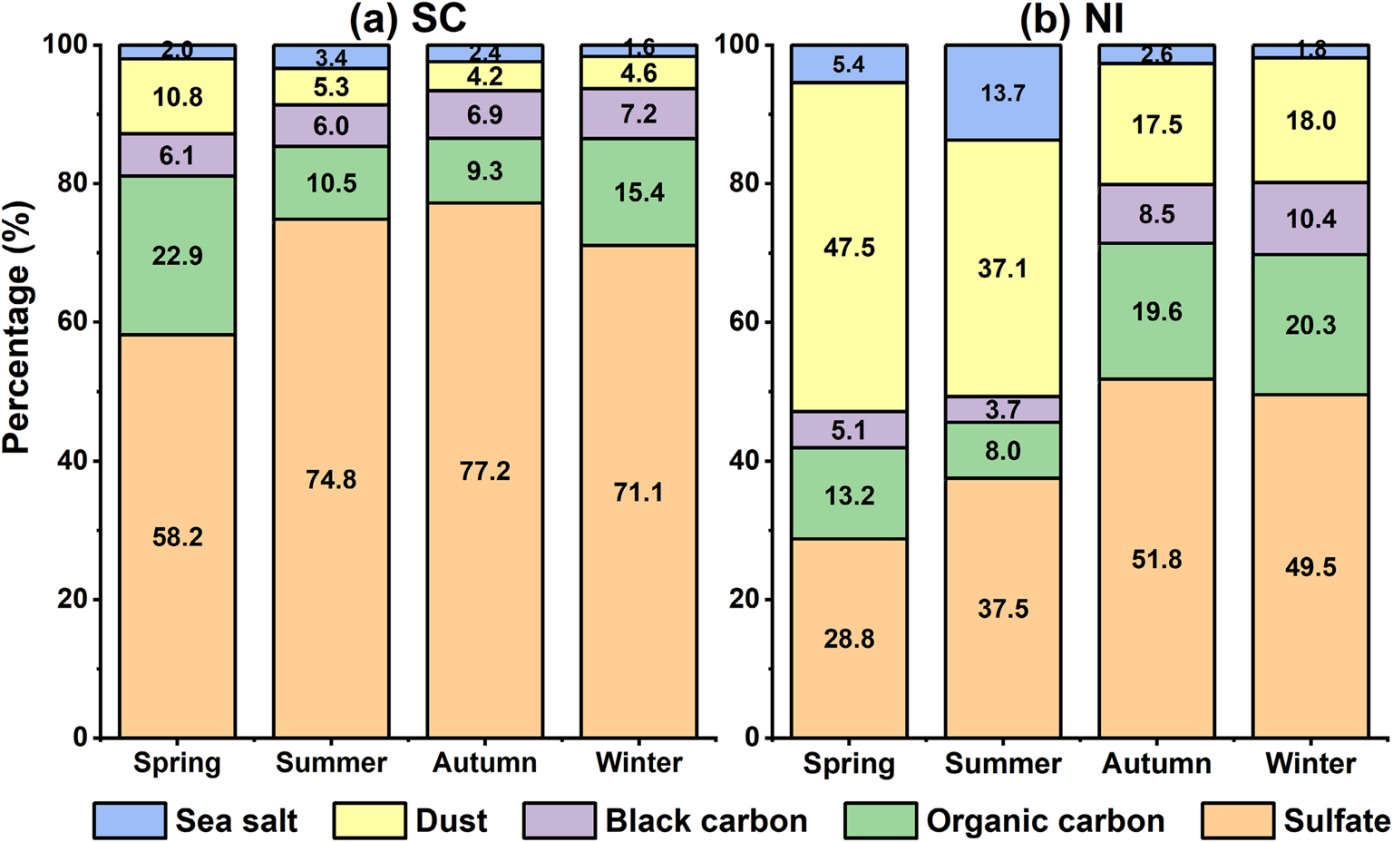
The percentages of the AOD of five aerosol types to the total AOD in (**a**) the SC and (**b**) the NI in the four seasons (Unit: %).

The percentages of AOD of various types of aerosols including sulfate, dust, black carbon, organic carbon, and sea salt to the total AOD are calculated using the MERRA-2 reanalysis products in the four seasons during the same period of time (Fig. 8). It is found that the percentages of different aerosol types vary in different seasons and there exists clear regional features in the SC and NI. In the SC region, the sulfate aerosol is the predominant contributor (58–77%) to the total AOD in every season. The percentages of dust and organic carbon aerosols are highest in spring and are lowest in autumn, while the black carbon and sea salt aerosols maintain relatively stable contribution of 6–7% and 2–3%, respectively.

For the NI region, the dust aerosol is another important regulator of AOD in addition to the sulfate aerosol. In certain season, the percentage of dust is close to (summer), or even surpasses the percentage of sulfate (spring). The other aerosol components also vary with seasons and exhibit some local features such as the relatively higher percentages of black carbon and organic carbon aerosols in autumn and winter, as well as the much higher percentage of sea salt aerosols in summer. Figures S8 and S9 further show the seasonal variation of the AOD distributions for sulfate and dust aerosols. Contrary to the SC where the high sulfate AOD center shifts from the northeast SC in summer to the central SC in the other seasons, the high sulfate AOD center in the NI remains mostly constant around the southern edge of the Himalayas ranges. Dust aerosol is mostly present in the northern SC in spring while the dust AOD is low in the other seasons. The Thar desert however provides ample dust aerosols and influences the northern NI in every season with the largest impacts being in the spring and summer. Apparently, the relative importance of sulfate and dust aerosols are different in the northwest and northeast of NI.

## Discussion

In this study, we focus on analyzing the influences of clouds and aerosols on the solar photovoltaic potential over the southern China and northern India, where very similar geographic location, population density, and solar energy demand but very different solar PV potentials are found. By using the CERES SYN1deg data products from 2003 to 2019 and the PVLIB-Python package, the plane-of-array-irradiances under different conditions are estimated, and the impacts of clouds and aerosols are also quantified as aerosol effect, cloud effect, percentage of POAI reduction by aerosols, and percentage of POAI reduction by clouds, respectively. The variations of cloud effect are found to be associated with the co-variations of cloud fraction and optical depth. The aerosol effect is closely related to the specific aerosol types and the unique optical properties.

The POAI in the SC and NI regions not only has different spatial distributions but also exhibits diverse temporal variations, with the former being remarkably lower than the latter except in summer. It is found that Guizhou and Chongqing in the western SC are the areas with the lowest POAI in the SC, while the Rajasthan state in the northwest of NI is where the highest POAI is found in the NI. The difference in POAI between the two regions is the largest in March and the lowest in August. The different cloud and aerosol conditions within the two regions are considered the most important factors to explain this phenomenon.

Given that the CERES SYN1deg data set provides a complete set of surface shortwave diffused and direct radiative fluxes under the pristine, clear-sky, all-sky, and all-sky-no-aerosol cases, it is convenient to separate and quantify the effects of clouds and aerosols on the solar PV potential. The annual averaged results show that clouds reduce the POAI by 2.67 kWh/m^2^/d in the SC and by 1.47 kWh/m^2^/d in the NI, while the reductions due to aerosols are 0.9 kWh/m^2^/d in the SC and 1.57 kWh/m^2^/d in the NI, respectively. It is also clear that aerosols and clouds have different relative importance in different seasons at the different locations. Contrary to the SC where the cloud effect is always much stronger than the aerosol effect, the impacts of aerosol and cloud are comparable in the NI with the former frequently larger than the latter except for summer.

By examining the cloud properties over the two regions, it is found that the SC region has nearly twice of the cloud fraction for the NI region except for summer, and the contrast of COD is also high between the two regions. It is further found that the middle clouds contribute the most to the CF and COD in the SC. The monthly changes in CF and COD correspond well with the peaks and lows of POAI in the SC and NI, which indicates that cloud is still the largest modulator of surface solar radiation. It is interesting to note that the diurnal variations of cloud properties could be another contributing factor to the different cloud effects between the two regions. As the cloud optical depth quickly increases after sun rise and remains high during daytime with the cloud fraction keeping at ~ 0.7 in the SC, much lower cloud fraction (~ 0.4) and much lower cloud optical depth are found in the NI.

Another interesting point is that although the aerosol optical depth over the SC is apparently larger than that over the NI, the aerosol impacts on the POAI are similar in magnitude over the two regions. The aerosols mostly composed of dust and sulfate over the NI are found to be nearly three times more efficient in reducing POAI than the counterpart which is mainly composed of sulfate over the SC. It should be noted that we didn’t consider the aerosol-cloud interaction which could be also contributing to the results of POAI distributions in this study. But our results clearly indicate that the aerosol effect and cloud effect are somehow correlated in both regions (Fig. S1), and previous study^49^ shows that sulfate aerosol particles can act as cloud condensation nuclei and increase cloud reflectivity.

## Conclusions

This study quantifies the different impacts of clouds and aerosols on the solar photovoltaic potentials for various seasons over the northern India and southern China. The differences in the cloud fraction and cloud optical depth, including their distinct diurnal variations, are found to be the major reasons for the large contrast of POAI reductions by clouds between the two regions. The aerosol components with varying capacities of solar radiation extinction are expected to explain the phenomenon of why higher aerosol optical depth results in lower aerosol impacts in southern China. Our findings are helpful for taking the initiatives to mitigate the adverse impacts of aerosol and cloud, and for further exploring the solar PV potentials in the specific regions.

## Supplementary Information

Supplementary Information.

## Data Availability

The CERES SYN1deg data set can be accessed online at https://ceres.larc.nasa.gov/. The MERRA-2 data set is available at https://disc.sci.gsfc.nasa.gov/datasets/.

## Code availability

The PVLIB-python package can be downloaded at https://pvlib-python.readthedocs.io/en/stable/index.html.
