# Supplementary material for: Diverse cloud and aerosol impacts on solar photovoltaic potential in southern China and northern India
Source: Sci Rep. 2022 Nov 16;12:19671. doi: 10.1038/s41598-022-24208-3 (PMC9669044; doi:10.1038/s41598-022-24208-3)
Supplement: Supplementary file 1 — Supplementary Information. [file 41598_2022_24208_MOESM1_ESM.docx]

Supporting Information for

**Diverse Cloud and Aerosol Impacts on Solar Photovoltaic Potential in Southern China and Northern India**

Jiangyan Yang^1^, Bingqi Yi^1*^, Shuai Wang^1^, Yushan Liu^1^ and Yuxiao Li^1^

^1^*School of Atmospheric Sciences and Guangdong Province Key Laboratory for Climate Change and Natural Disaster Studies, Sun Yat-sen University, and Southern Marine Science and Engineering Guangdong Laboratory (Zhuhai), Zhuhai, China.*

* Corresponding author: Dr. Bingqi Yi, yibq@mail.sysu.edu.cn

This PDF file includes 9 figures.


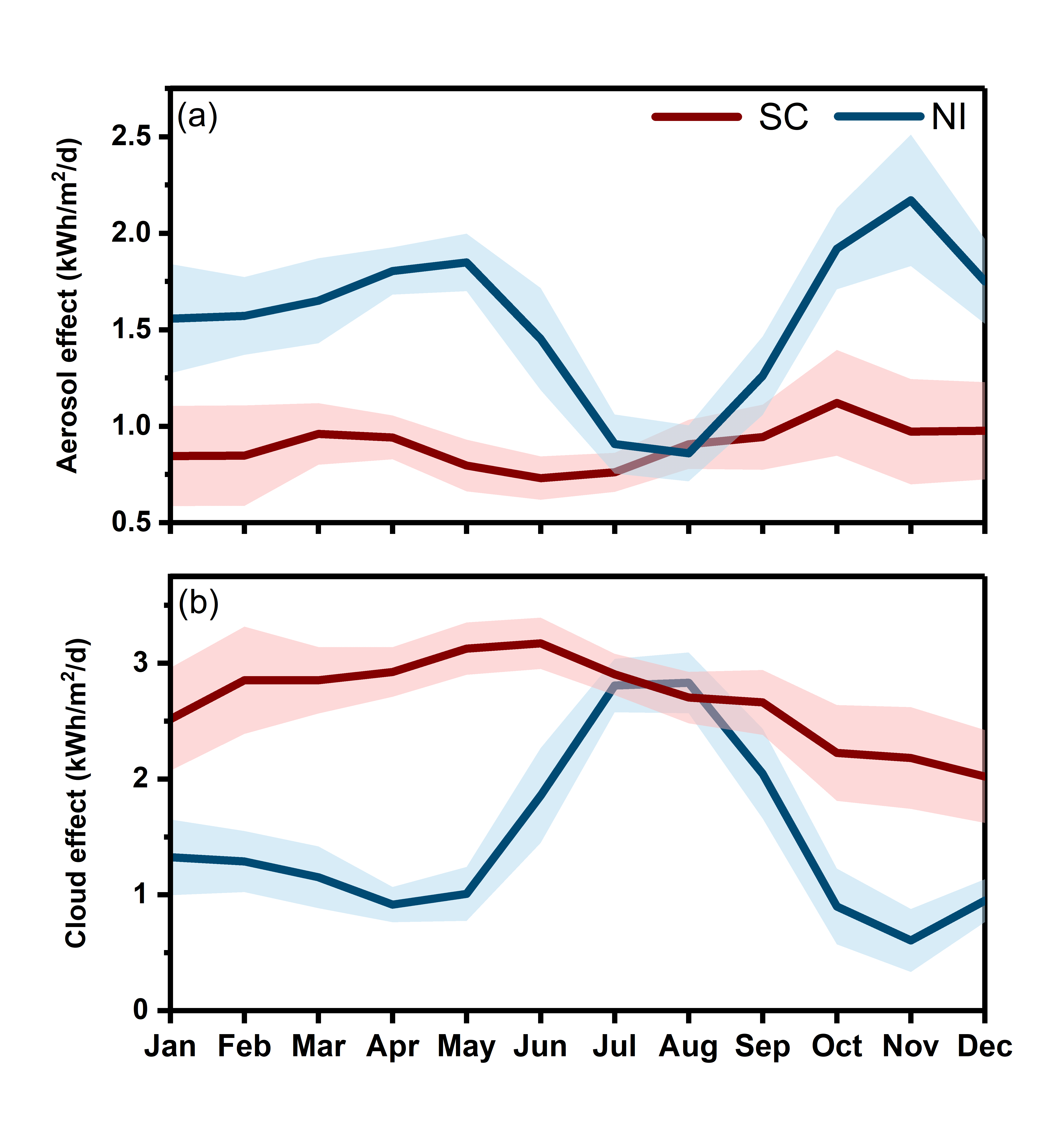


**Fig. S1.** Monthly averaged variations of the (a) aerosol effect and (b) cloud effect over the SC and NI regions from 2003 to 2019.


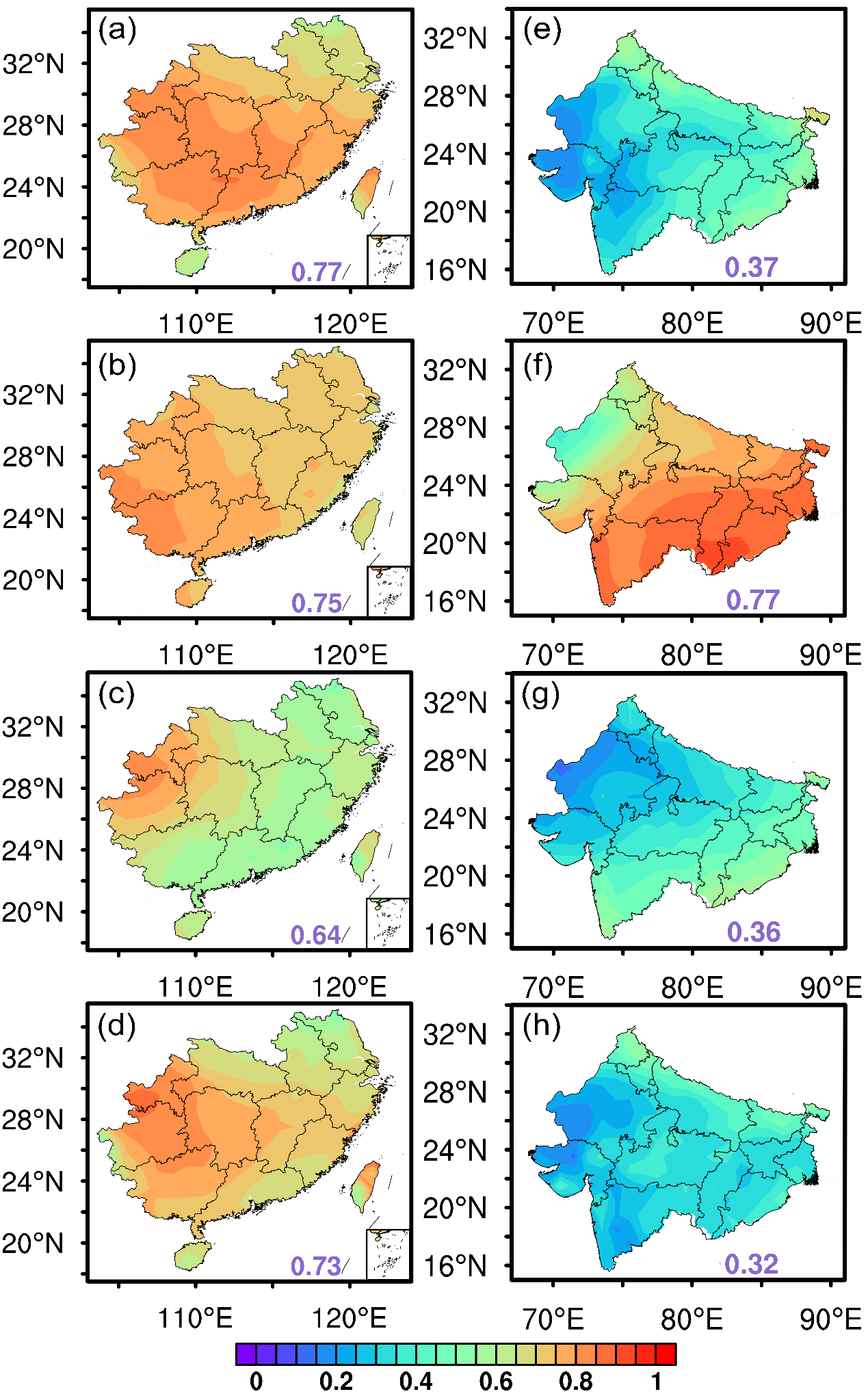


**Fig. S2.** Seasonal averaged cloud area fraction from 2003 to 2019 over the SC (left column) and the NI (right column). (a, e) spring (March-April-May); (b, f) summer (June-July-August); (c, g) autumn (September-October-November); (d, h) winter (December-January-February). The map was generated by ESRI ArcGIS 10.5 software available at the ESRI website (https://www.esri.com/en-us/arcgis/products/arcgis-platform/overview). The administrative boundary shapefile for southern China is available at the RESDC website (https://www.resdc.cn/) and the administrative boundary shapefile for northern India is available at the GADM website (https://gadm.org/download_country.html).


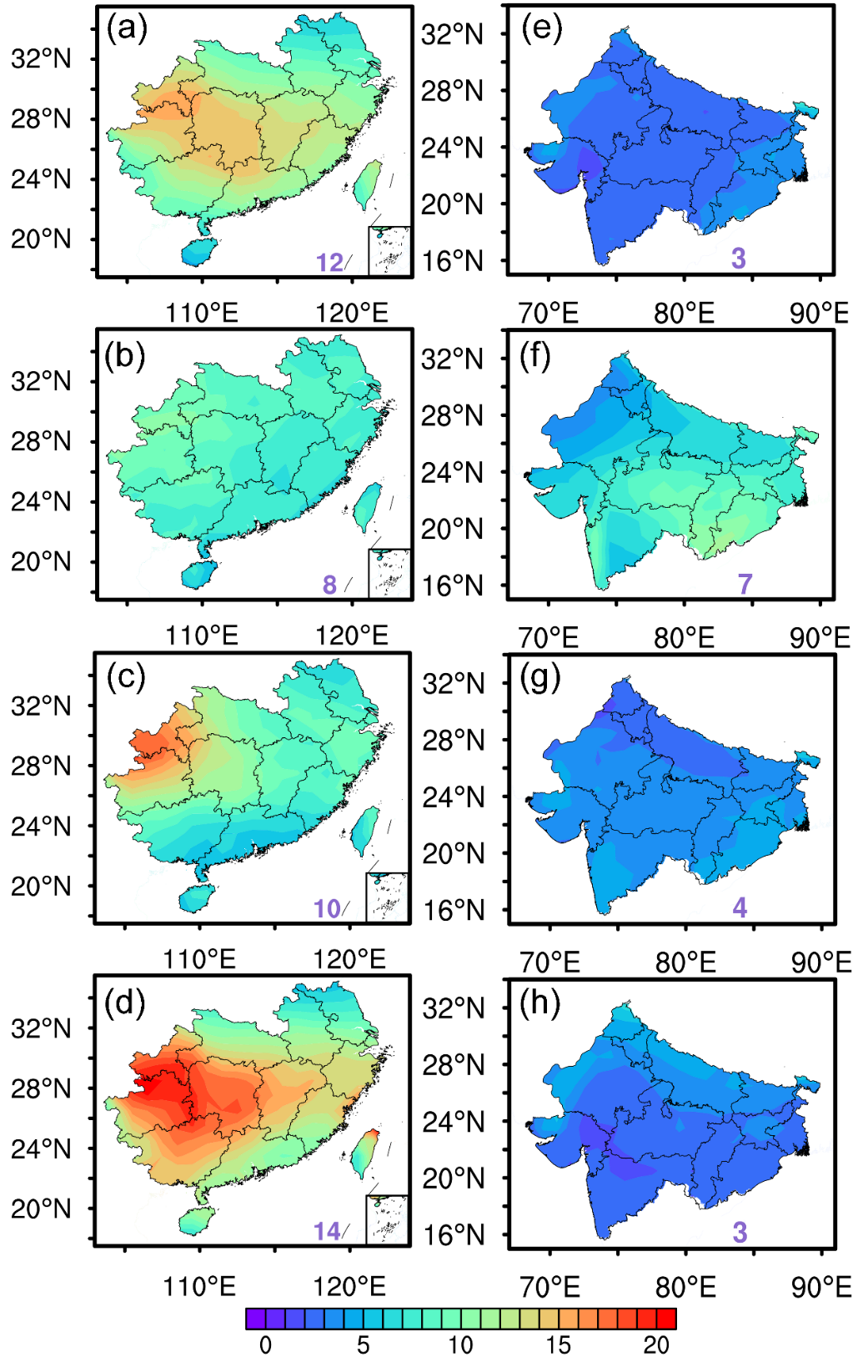


**Fig. S3.** The same as Fig. S2, but for the cloud optical depth. The map was generated by ESRI ArcGIS 10.5 software available at the ESRI website (https://www.esri.com/en-us/arcgis/products/arcgis-platform/overview). The administrative boundary shapefile for southern China is available at the RESDC website (https://www.resdc.cn/) and the administrative boundary shapefile for northern India is available at the GADM website (https://gadm.org/download_country.html).


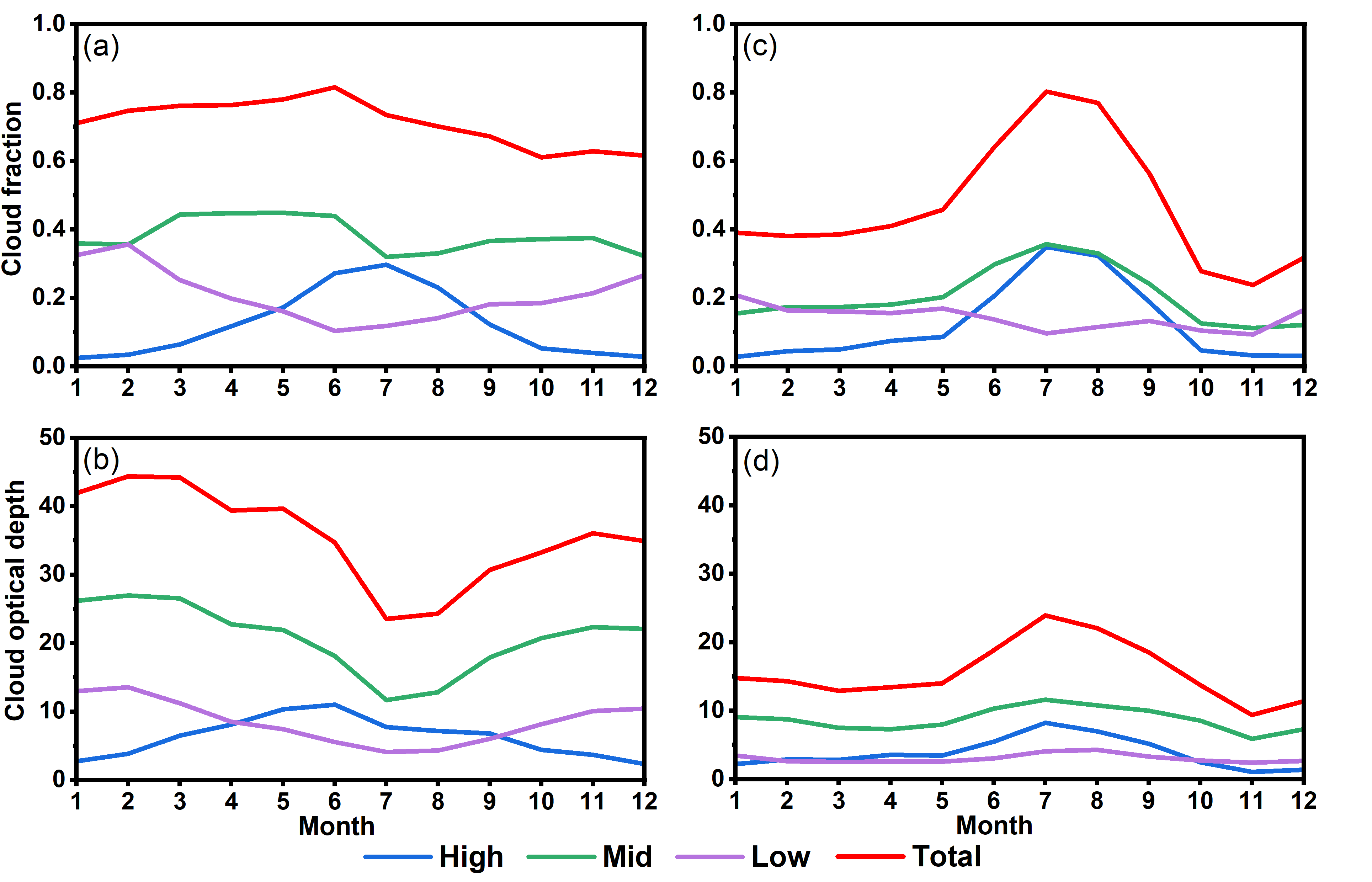


**Fig. S4.** Monthly averaged variations of cloud area fraction (a, c) and cloud optical depth (b, d) for high, mid, low clouds and total clouds from 2003 to 2019 over the SC (a, b) and NI (c, d).


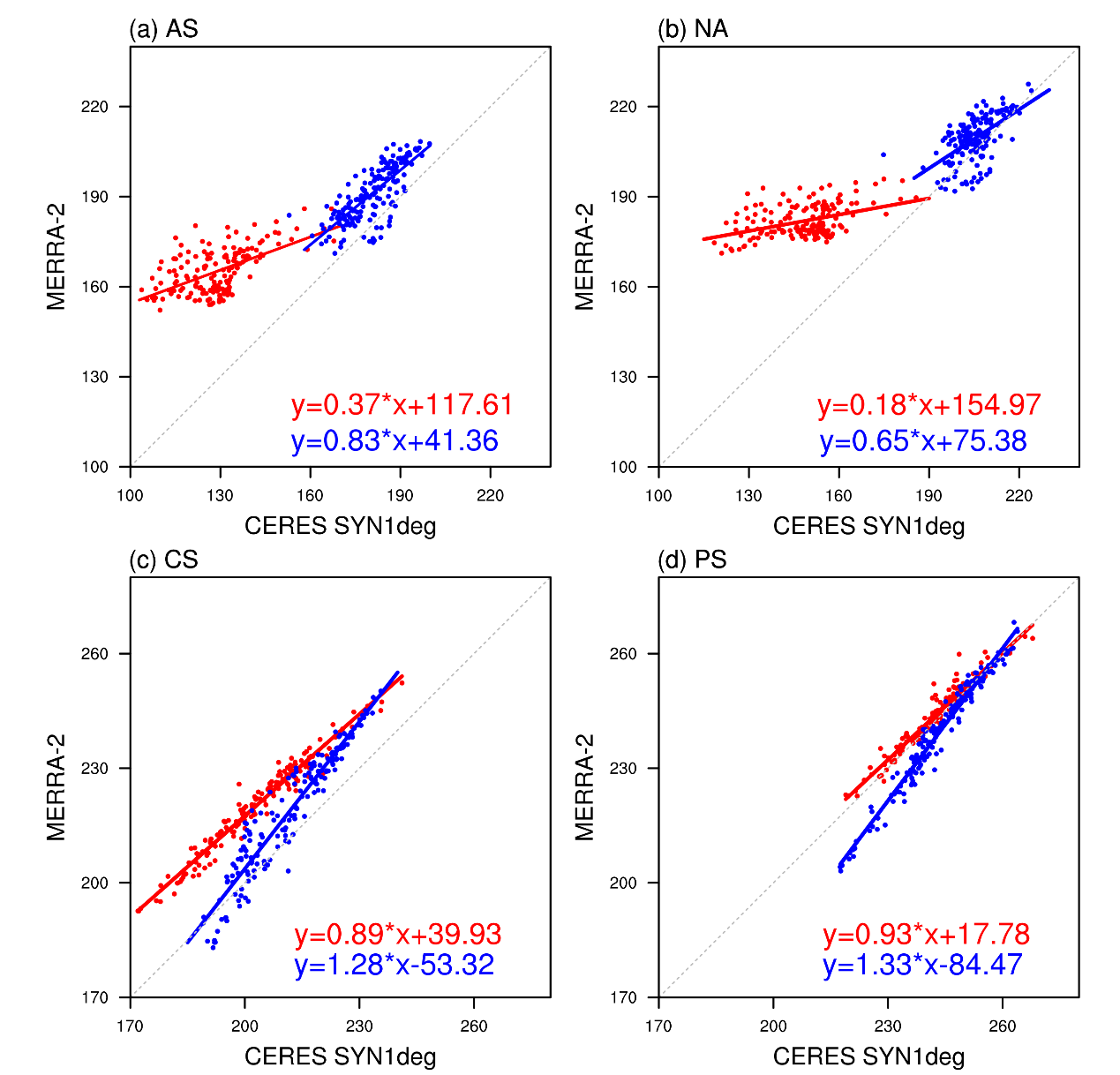


**Fig. S5.** Scatter diagrams of the annual averaged surface shortwave radiant fluxes of the CERES-SYN1deg and MERRA-2 data sets over the SC (red) and NI (blue) regions. (a) AS: all-sky condition; (b) NA: all-sky no aerosol condition; (c) CS: clear-sky condition; (d) PS: pristine condition. The linear regression between CERES and MERRA-2 are shown.


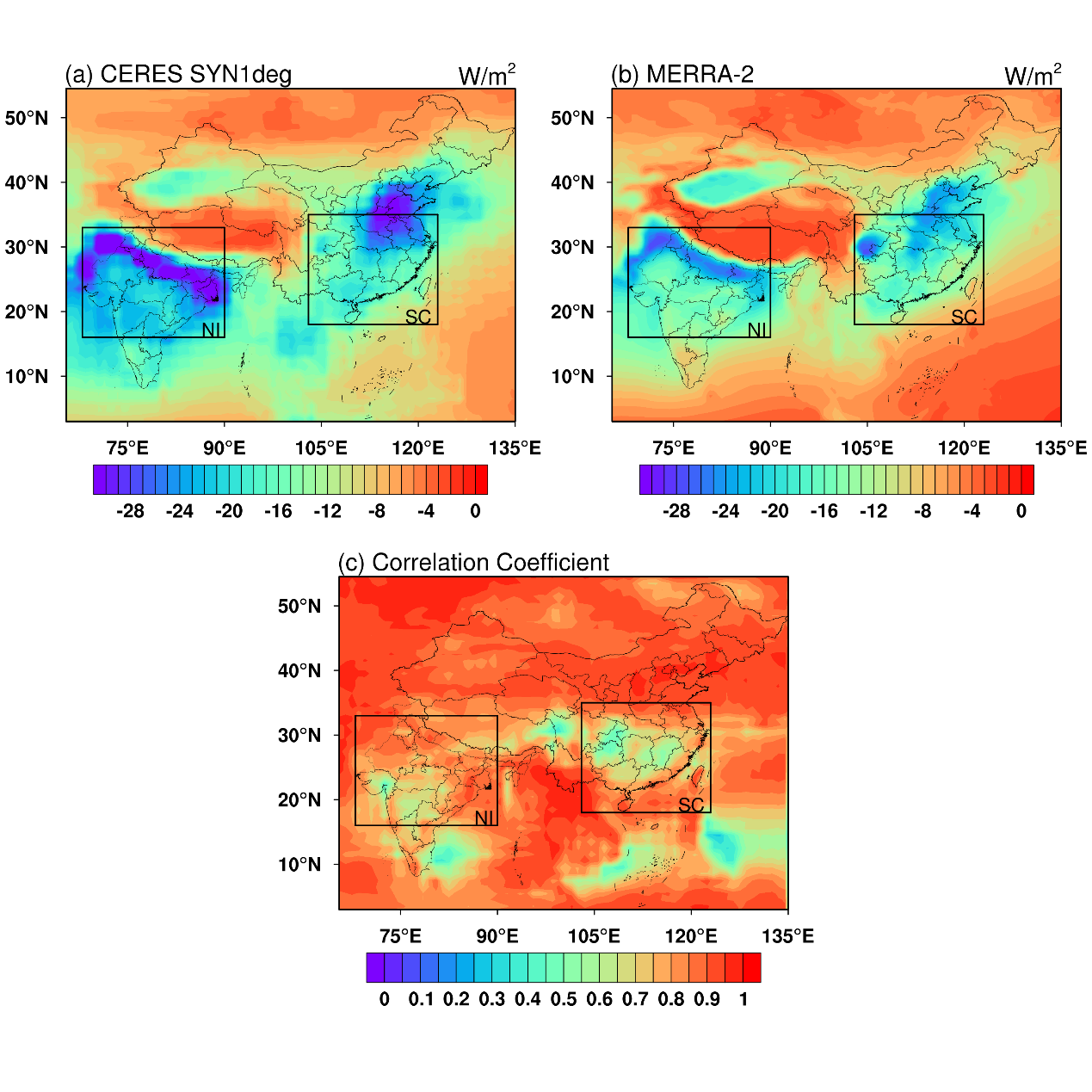
**Fig. S6.** Comparison of the annual averaged shortwave aerosol radiative forcing at the surface between the CERES-SYN1deg and MERRA2 data sets. (a) CERES-SYN1deg; (b) MERRA-2; (c) the correlation coefficients between the two data sets. The map was generated by ESRI ArcGIS 10.5 software available at the ESRI website (https://www.esri.com/en-us/arcgis/products/arcgis-platform/overview). The administrative boundary shapefile for southern China is available at the RESDC website (https://www.resdc.cn/) and the administrative boundary shapefile for northern India is available at the GADM website (https://gadm.org/download_country.html).


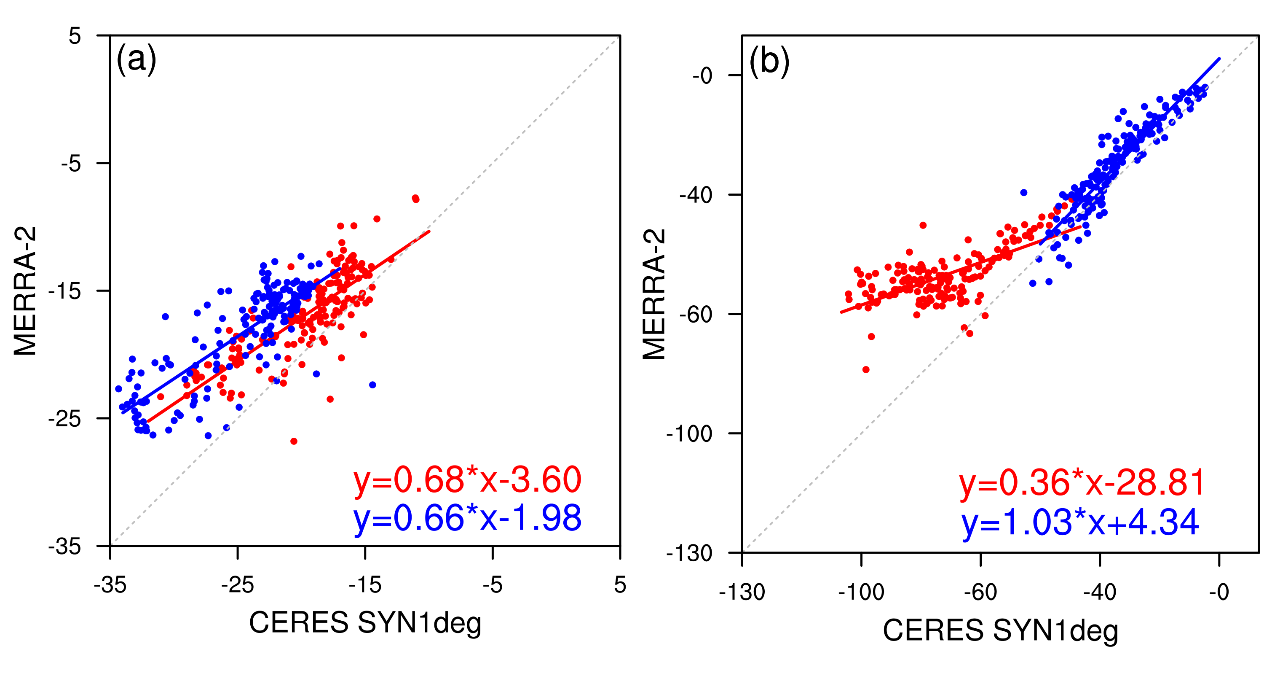


**Fig. S7.** Scatter diagrams of the annual averaged surface (a) aerosol radiative forcing and (b) cloud radiative forcing of the CERES-SYN1deg and MERRA-2 data sets over the SC (red) and NI (blue) regions. Unit: W/m^2^. The linear regression between CERES and MERRA-2 are shown.


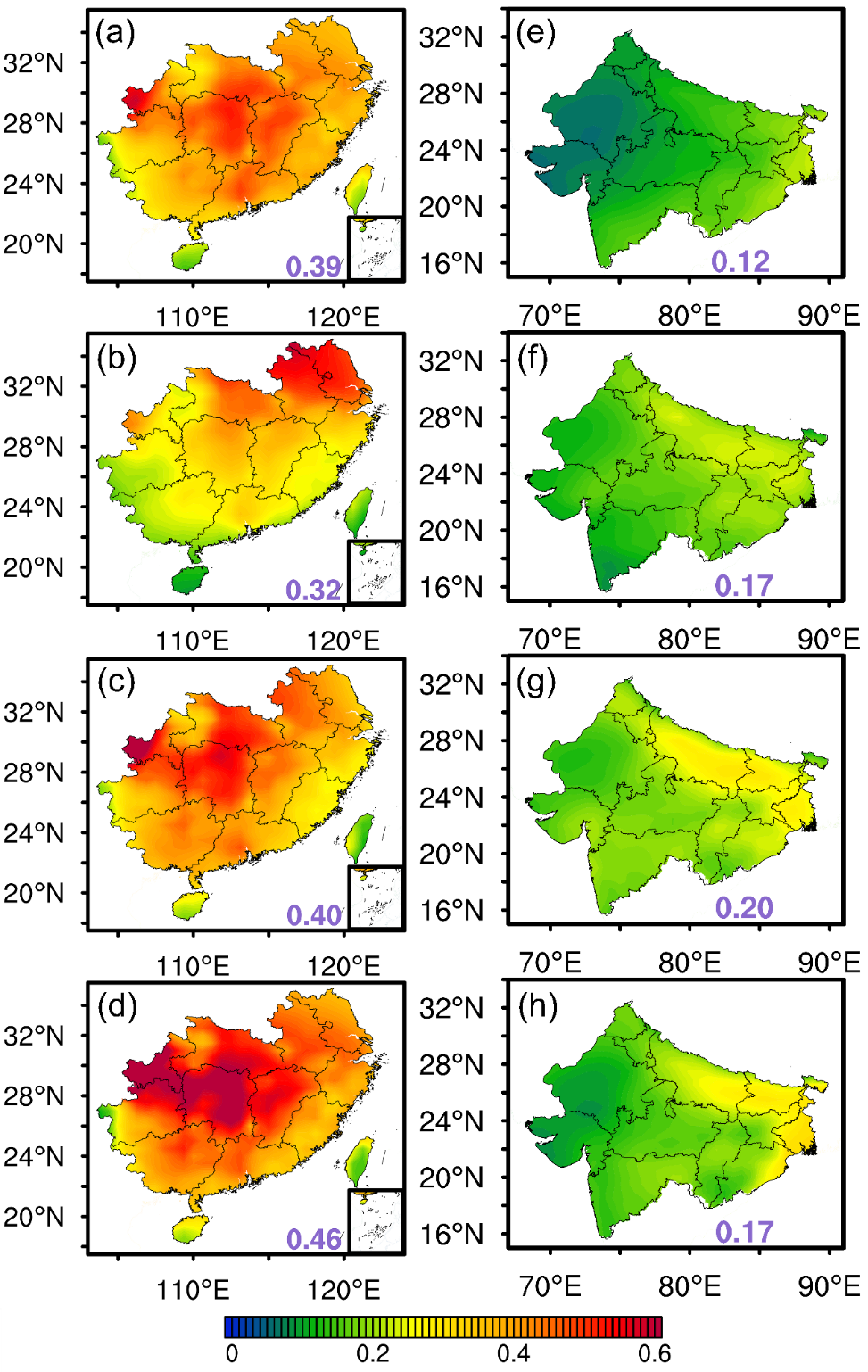


**Fig. S8.** Spatial distribution of seasonal averaged AOD for sulfate aerosol from 2003 to 2019 over the SC (left column) and the NI (right column). (a, e) spring (March-April-May); (b, f) summer (June-July-August); (c, g) autumn (September-October-November); (d, h) winter (December-January-February). The map was generated by ESRI ArcGIS 10.5 software available at the ESRI website (https://www.esri.com/en-us/arcgis/products/arcgis-platform/overview). The administrative boundary shapefile for southern China is available at the RESDC website (https://www.resdc.cn/) and the administrative boundary shapefile for northern India is available at the GADM website (https://gadm.org/download_country.html).


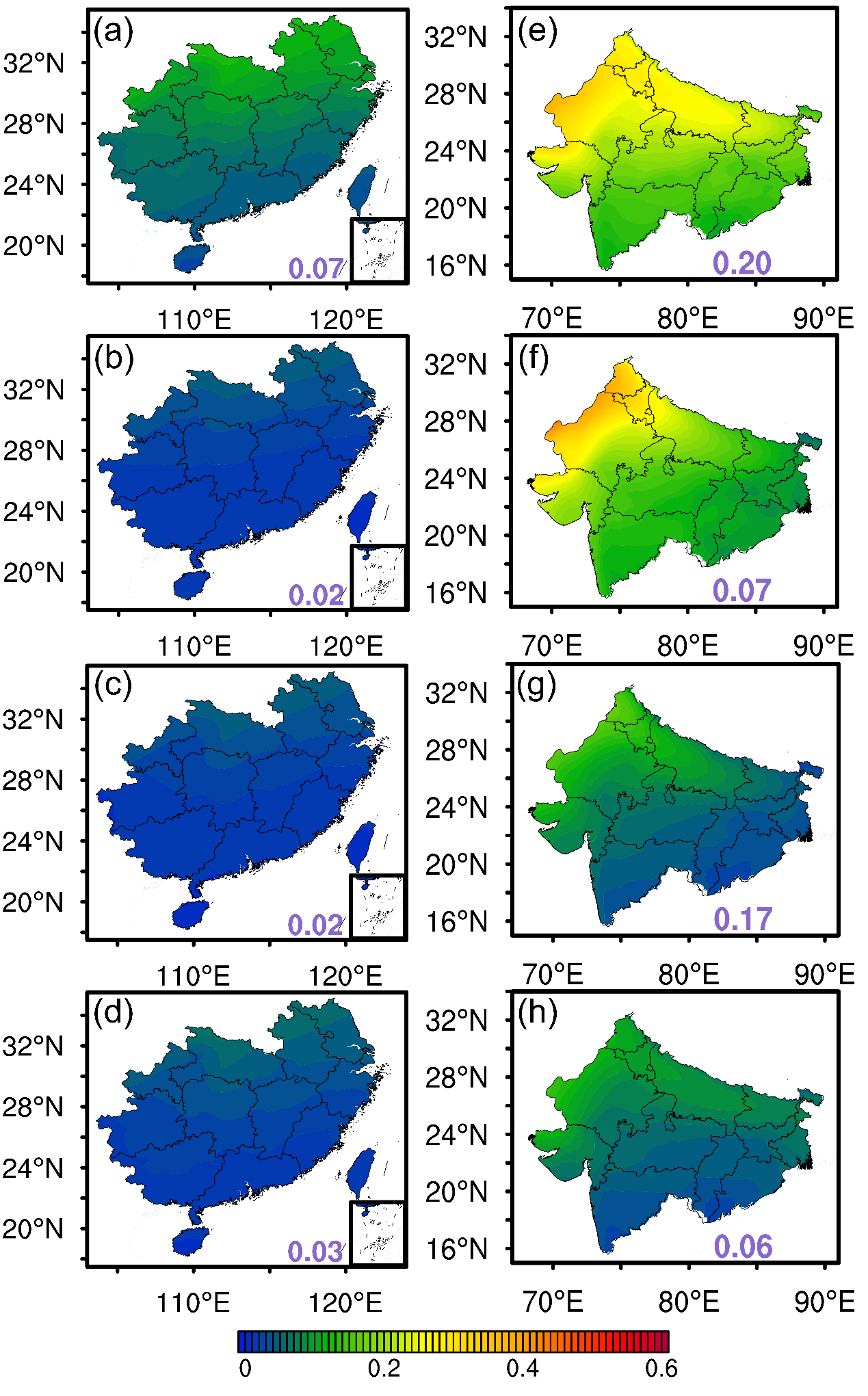


**Fig. S9.** Spatial distribution of seasonal averaged AOD for dust aerosol from 2003 to 2019 over the SC (left column) and the NI (right column). (a, e) spring (March-April-May); (b, f) summer (June-July-August); (c, g) autumn (September-October-November); (d, h) winter (December-January-February). The map was generated by ESRI ArcGIS 10.5 software available at the ESRI website (https://www.esri.com/en-us/arcgis/products/arcgis-platform/overview). The administrative boundary shapefile for southern China is available at the RESDC website (https://www.resdc.cn/) and the administrative boundary shapefile for northern India is available at the GADM website (https://gadm.org/download_country.html).
